# The underbelly of E-cigarette advertising: regulating online markets on social media platforms

**DOI:** 10.1186/s12954-024-01027-5

**Published:** 2024-05-29

**Authors:** Carmen C.W. Lim, Tianze Sun, Giang Vu, Gary C.K. Chan, Janni Leung

**Affiliations:** 1 National Centre for Youth Substance Use Research, The University of Queensland, St Lucia, QLD 4067 Australia; 2 NHMRC Centre of Research Excellence on Achieving the Tobacco Endgame, School of Public Health, Faculty of Medicine, The University of Queensland, Herston, QLD 4006 Australia; 3 School of Psychology, The University of Queensland, St Lucia, QLD 4067 Australia

**Keywords:** E-cigarettes, Nicotine vaping products, Social media

## Abstract

Australia prohibits the sale of nicotine-vaping products unless prescribed by medical practitioners. Significant policy reforms were announced on the 28th of November 2023 including a ban on single-use disposable vapes with and without nicotine, and the removal of the personal importation scheme. Despite stringent regulations, loopholes exist such that e-cigarette vendors are getting around it, and online markets provide a route to do so. We discuss strategies used by vendors to covertly market e-cigarettes online through social media. In this perspective, we highlight three proposed policies to strengthen social media regulations that may be feasible to implement. Our proposed strategies to regulate e-cigarette product listings on social media involve implementing robust age verification measures, enhancing the system for flagging and reporting prohibited content, and developing a more effective system to identify and flag content related to e-cigarettes.

## Background

A recent Cochrane review provided a higher quality of evidence that nicotine vaping products (NVPs) can be an effective smoking cessation aid than nicotine replacement therapies [1]. Despite being a safer alternative than traditional cigarettes owing to fewer combustion by-products, the global regulatory laws regarding the sales of NVPs differ, reflecting the diverse governmental responses to the escalating threat of youth vaping. In Australia, all NVPs are prescription-only medicines across all Australian states and territories as of October 2021 [2]. It is illegal to import, buy, or sell NVPs without a valid prescription or permit [2]. In stark contrast, New Zealand allows the sale of NVPs but imposes stringent regulations to protect public health. Specifically, New Zealand only permits the sale of reusable products that meet strict safety requirements, including nicotine limits and child safety features [3]. In both Australia [4] and New Zealand [3], any form of NVP advertising is strictly prohibited, however, the existence of a flourishing black market [5] and the high prevalence of youth vaping [6] indicate regulatory gaps.

**Fig. 1:**
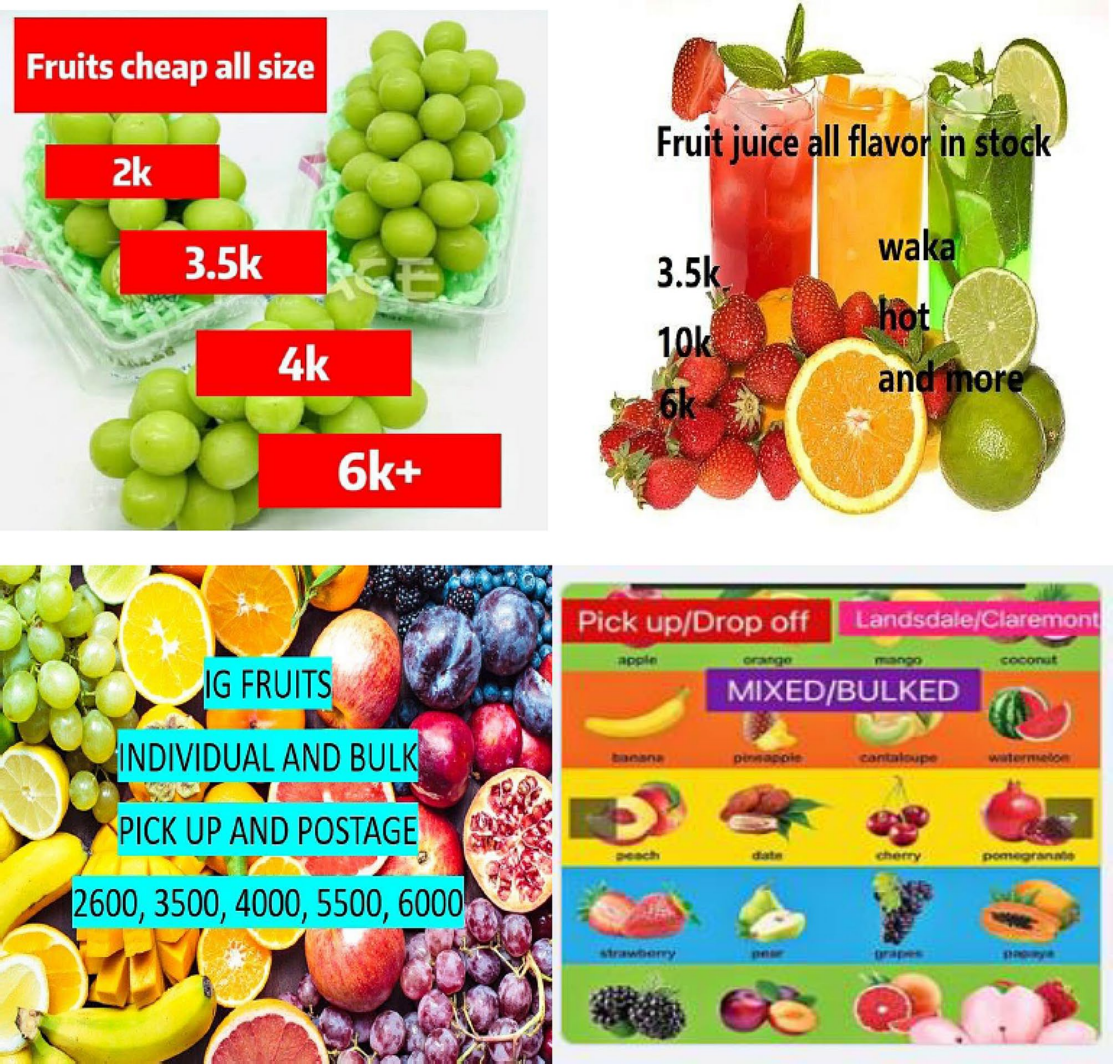
Examples of e-cigarette listings on Facebook Marketplace (October 2023)

E-cigarettes are readily accessible online [5] due to the cross-border nature of social media. Although online marketplace platforms prohibit nicotine product listings [7], significant loopholes exist such that social media users are still able to purchase these products online. In October 2023, we conducted an online search and identified e-cigarette advertisements on Facebook Marketplace, an online marketplace platform. Among some tactics used to evade detection, possibly as a response to regulatory pressures include the use of: 1) *Misleading pictures and texts* - instead of overtly showing a picture of a vape, vendors post other images (e.g., fruits and juices) and used ambiguous phrases (e.g., fruit juices, all flavours in stock), which alludes to the vapes’ flavours (Fig. 1); 2) *Code words* – vendors used numbers on their images (e.g., 3500) as discreet indicators of the number of puffs on a vape pen. The term ‘bulk’ is used to subtly convey that bulk sales are available, appealing to those wanting to purchase in larger quantities. In addition, some listings also prompted consumers to direct message regarding bulk purchases and doorstep delivery.

Exposure to e-cigarette marketing on social media, along with a lack of age verification enforcement online [8] is associated with lower risk perceptions, and increased susceptibility to and subsequent e-cigarette use [9]. The recent announcement made by the Australian government on vaping, while comprehensive, did not specifically address the role of social media in influencing use, particularly considering that a significant portion of the youth demographic actively uses social media. Outside of Australia, other jurisdictions have taken steps to regulate e-cigarette marketing. For example, in 2021, the U.S. Food and Drug Administration (FDA) required all e-cigarette retailers to provide detailed information about their social media practices (data on advertising plans, target audiences, and actions taken to restrict youth exposure to e-cigarette content online) [10]. The FDA also sent warning letters to companies that employed paid social media influencers to promote flavoured nicotine solutions to their online followers [11]. 

### Proposed policy options

Efficient policing on social media platforms is challenging, but not impossible. Here, we propose three feasible policy options for consideration: mandating social media platforms to: (i) strengthen their age verification systems (e.g., using proof through official ID) to prevent underage users from accessing and purchasing e-cigarettes; (ii) make the reporting process for prohibited content more user-friendly and responsive, to ensure that users feel that their concerns are being taken seriously; and (iii) train their system to detect, flag, and remove content related to the sale of prohibited items like NVPs. With advancements in Artificial Intelligence, algorithms can be trained to recognise not only explicit mentions but also the coded language, imagery, and patterns of behaviour typical of illicit sellers.

Authorities have the power to regulate marketing-related activities when they reach inside their borders [12]. A notable example of such regulatory success is the Australian government’s approach to managing the online content of Ozempic, a prescription medication intended for diabetes treatment that had been extensively promoted on social media platforms for weight loss [13]. This case illustrates how government can effectively enforce laws against unauthorised promotions on social media platforms, setting a precedent for similar actions against NVP promotions. This can involve measures such as fines, restrictions, or legal action against companies that fail to comply with national regulations.

## Conclusion

Along with the recent suite of announcements made by the Australian Federal Government, it is important to have in place scalable and effective content moderation to mitigate the negative effects of the black market. Our proposed solutions to regulate NVPs content on social media include the need for a more robust age verification, flagging and reporting of prohibited content more user-friendly and responsive, and for a more robust system to detect and flag content related to prohibited items such as, NVPs. Each of these solutions has its own set of challenges and limitations, but a combination of these strategies could provide a more robust and dynamic approach to policing social media for the sale and marketing of NVPs.

## Data Availability

No datasets were generated or analysed during the current study.
